# Statistical inferences under the Null hypothesis: common mistakes and pitfalls in neuroimaging studies

**DOI:** 10.3389/fnins.2015.00018

**Published:** 2015-02-19

**Authors:** Jean-Michel Hupé

**Affiliations:** Centre de Recherche Cerveau et Cognition, Université de Toulouse and Centre National de la Recherche ScientifiqueToulouse, France

**Keywords:** statistical inference, Null hypothesis significance test, random field theory, permutation tests, false discovery rate

## Abstract

Published studies using functional and structural MRI include many errors in the way data are analyzed and conclusions reported. This was observed when working on a comprehensive review of the neural bases of synesthesia, but these errors are probably endemic to neuroimaging studies. All studies reviewed had based their conclusions using Null Hypothesis Significance Tests (NHST). NHST have yet been criticized since their inception because they are more appropriate for taking decisions related to a Null hypothesis (like in manufacturing) than for making inferences about behavioral and neuronal processes. Here I focus on a few key problems of NHST related to brain imaging techniques, and explain why or when we should not rely on “significance” tests. I also observed that, often, the ill-posed logic of NHST was even not correctly applied, and describe what I identified as common mistakes or at least problematic practices in published papers, in light of what could be considered as the very basics of statistical inference. MRI statistics also involve much more complex issues than standard statistical inference. Analysis pipelines vary a lot between studies, even for those using the same software, and there is no consensus which pipeline is the best. I propose a synthetic view of the logic behind the possible methodological choices, and warn against the usage and interpretation of two statistical methods popular in brain imaging studies, the false discovery rate (FDR) procedure and permutation tests. I suggest that current models for the analysis of brain imaging data suffer from serious limitations and call for a revision taking into account the “new statistics” (confidence intervals) logic.

## Foreword

The present text was not written by a statistician or a developer of MRI analysis, but by a “statistics-aware” MRI end user. It may contain questionable statements or approximations. More authoritative references are provided. On the other hand, most of it that is correct should be obvious for a specialist, who may not learn anything new. However, many MRI users may find here matter of reflection. MRI is more complicated than what efficient and widely available analysis programs may suggest. Critically, many serious papers using sophisticated techniques contain errors involving the simple and basic logic of statistical inference. Most of these errors have already been denounced in other articles. Here I made a short list of those as well as pitfalls of MRI analysis. Readers may consult the Appendix of the companion review paper on synesthesia to read about precise examples (Hupé and Dojat, 2015). I hope that sharing the understanding achieved by a once naive MRI end user would benefit other MRI end users.

The goal of this paper is not to provide new guidelines, new statistical recipes or any kind of authoritative reference. The “tools” used here should be shared by any scientist: common sense, logical reasoning and thought experiments. The minimal knowledge about statistical inference and MRI analysis, when required, is also reminded.

The first part of this paper describes therefore what I consider as the very basics of statistical inference, and what I understood of Null Hypothesis Significance Tests (NHST). The second part describes when such statistical inference was not correctly applied in MRI studies. The list may not be exhaustive: it contains the errors we found in our review of the literature on synesthesia. The third part describes the main analysis pipelines used in MRI studies. Again, the list is not exhaustive because based only on the literature we reviewed. Even though it does not include the latest developments, this part does describe critical steps and pitfalls that all studies have to face. The idea is certainly not to tell that these pipelines are wrong, or to tell which method is the best. The idea is to highlight or to remind fundamental difficulties that these methods tried to solve. Approximations or unverifiable assumptions may well be appropriate for certain studies: making them or not is the responsibility of the researcher. I hope that trying to clarify them would help researchers taking the best decisions.

## Background: statistical inference and NHST

### Statistical inference

Empirical investigations are based on statistical inference, even before computing any kind of statistical test: one wants to draw general conclusions (the population) based on a limited set of observations (a sample). Biological and psychological measurements are noisy. Most quantitative measures assume a model of the form: empirical observation = true value + error. A single observation is meaningless when the possible magnitude of the error is unknown. Estimating the true value, therefore, requires making several observations “everything else being equal.” In statistical terms, observations need to be considered as “independently and identically distributed (i.i.d.)” random variables. This hypothesis depends on the empirical design and is often difficult to prove or control entirely (the state of a subject in the scanner can never be the same at the different times when the BOLD signal is measured). When the sources of measurement errors are multiple, errors typically follow a Gaussian distribution (random noise) and their sum converges toward zero. The average of observations is then a good (unbiased, convergent) estimator of the true value. But there are cases when computing the average of observed values is not correct or not very informative about the population, for example when the distribution of measures is not symmetrical around its mean, like for a Lognormal distribution. In that case, observed for bounded measures like, often, response times, an appropriate summary measure is the average of the logarithm of the measures, because after data transformation the errors follow a Gaussian distribution^1^.

The normal distribution of errors, assumed in most cases, is difficult to verify unless many observations are available. A critical question is the minimum number of observations needed for any statistics (including summary statistics). Summary statistics and tests can be computed with even a very small number of observations. However, estimating the validity of the assumptions supporting statistical inference is impossible with small numbers. Non-parametric, randomization or bootstrap tests are theoretically more valid because they rely on fewer assumptions. For example, the results of a permutation test are *theoretically* valid whatever the sample size as long as the assumption of exchangeability is valid. However, these results may not be *empirically* valid for small numbers because too few measurements may not be representative enough of the population: one can never exclude the possibility that something unnoticed went wrong with one measure or subject. Any statistical measure should not be critically dependent on any single measure. Crossvalidation methods could be systematically applied for small samples. You may, for example, remove one subject in your analysis to check if the results still hold (leave-one-out procedure, jackknife) or split your sample in two (half-split reliability). But cross-validation is only possible when you have enough data (see below). A related problem due to small samples is known in statistics as overfitting, which leads to an inflation of observed significance and effect size when a few measures drive most of the effect. Overfitting can be overcome with cross-validation methods (for example by reporting the minimum effect size or larger *p*-value measured when removing any one measure or subject). Removing outliers based *only* on the distribution, without prior knowledge (or documented assumption) on the data distribution, is however not acceptable practice (fitting of the data to the statistical model; if the data distribution does not conform to the validity conditions of the model, a better model should be found^2^).

### The ill-posed logic of NHST: Type III errors

NHST correctly compute the probability of observing an empirical value (the sample statistics) under the assumption that the Null hypothesis is true. When this probability is low, one may decide to take the risk of rejecting the Null hypothesis. If the Null is true, this risk corresponds to a Type I error. Such reasoning allows neither computation of the *probability* of being wrong when not rejecting the Null Hypothesis (which is a Type II error), nor computation of the *probability* of being wrong when rejecting the Null (Cohen, 1994; Kline, 2004). This is because the computed probabilities concern the random samples given a true population value, which is never known. What we are interested in is the probability of the population value given the observed value in a given sample. Such computation is not possible without knowing the priors (Bayes theorem). As phrased by Killeen (2005), when a *p*-value is below 0.05 (arbitrary, conventional threshold), one can only be “surprised.” However, publication standards enforce that almost only so-called “significant” results be published, and that “significant” rejections of the Null hypothesis be considered as “proven.” Such strong emphasis on “significance” is problematic with MRI studies (in particular), where controlling everything is not possible, like for example the exact matching of subjects when comparing groups (empirical groups of synesthetes and controls may differ on things other than synesthesia, for example, motivation) or the exact balance between stimuli (attention bias, if for some reason one condition looks more interesting than the other). In other words, in a given experiment small differences of no interest for the question at stake always exist: the Null hypothesis is never true. False premises (the Null hypothesis) “lead to conclusions that may be logically consistent but empirically invalid” (Killeen, 2005), what is called a Type III error (correctly rejecting the Null hypothesis but for the wrong reason). Of course such differences will not be reliable if the paradigm or procedure changes, and, therefore, not replicable. But they will generate “significant” effects when increasing the number of measures (e.g., Cohen, 1994). Indeed, if you consider the Cohen measure of effect size *d*′ = (μ1 − μ2)/*sd* (difference of means over the standard deviation), you obtain *t* = *d*′^*^sqrt(*n*), meaning that any weak effect becomes significant when increasing the number of measures *n*. This is an *empirical* law of statistics. Such consideration led Friston (2012) to recommend that MRI studies should not involve too many subjects lest very small, unreliable effects be published (the optimal number was between 16 and 32; note that this is still larger than in most studies on synesthesia considered in our review, but in most cases this number would produce underpowered, not reproducible, studies: Yarkoni et al., 2010; Button et al., 2013). But large samples may only be a problem when relying solely on a “significance” threshold. By increasing *n* you get a better estimation of your effect size, and this is what you want (Cumming, 2012; Ingre, 2013). Friston also argued that within small samples only effects large enough will be significant, and these are the effects we are most interested in, so “significant results from small samples should be taken more seriously than the equivalent results in oversized studies.” This logic is however faulty. In fact, if within small samples “significant” effects are indeed always *measured* as large, this is obtained with a large confidence interval. At *p* = 0.05, the 95% confidence interval of the true effect size includes zero, meaning that a very small effect, possibly due to sampling error, may easily be “significant” (and estimated as large) in a small sample (e.g., Christley, 2010), especially because of the inflation of observed significance and effect size in small samples (Yarkoni, 2009). Sampling error is inversely proportional to sampling size. In other words, larger samples are always better, and *p* < 0.05 is not a sufficient criterion to be surprised and doubt about the Null hypothesis (Johnson, 2013), because we already know that due to empirical constraints the Null hypothesis is never true (whatever the presence or not of any effect related to the design and tested question).

### The ill-posed logic of NHST: multiple comparisons

NHST compute the chance of observing values that deviate from a theoretical value, due to random sampling noise. Repeating tests at the 5% chance level guaranties observing some extreme values at least once (http://xkcd.com/882/). Voxel-based analysis in MRI requires to perform thousands of NHST, and, therefore, to adjust the individual statistical threshold accordingly to reduce the risk of making at least one error (correction for multiple comparisons in order to control the “family-wise error,” FWE, when considering the whole *family* of tests). Subjectively, it may yet still be surprising that a *p*-value equal to 0.001 and measured at a given voxel should be considered as non-significant (one chance over 1000) only because many more voxels were tested. Yet it should (Bennett et al., 2009). On the other hand the procedure to correct for multiple comparisons increases the risk of Type II error, which is the risk of not rejecting the Null hypothesis when, in fact, it is false. With limited power, a true effect at a given voxel may not be more “significant” than a random variation at another voxel. In MRI, these procedures are, therefore, often considered as “too conservative,” but, as pointed by Nichols (2012), “that's like saying a meter is too short. FWE is just a measure of false positive risk, a stringent one.” The correct way to decrease the type II error is to increase the sample size, not to increase the risk of false positives.

The crucial question is, in fact, the definition of the family of possible inferences to consider for a given question. For example, one may study only voxels in the visual cortex when measuring the response to visual stimuli. But when there is no obvious consensus for the definition of the family, this procedure looks arbitrary since the set size depends on the number of *observed* comparisons (the rest of the brain may have been recorded but not analyzed). Some statisticians, therefore, recommend “good practices” where researchers should tell in advance what comparisons they will make, to avoid deciding *post-hoc* what tests to include in their analysis. Yet such a practice would yield to at least paradoxical, if not absurd, consequences when, for example, two researchers with the exact same data set would reach different conclusions only because they had different hypotheses (Dienes, 2011); or if one of them, by being more ambitious and performing additional tests (maybe useful control tests), would not reach “significance” and, therefore, publication standards. This thought experiment suggests that we should consider the number of possible comparisons (the whole family) and not only the number of actual comparisons—which is also absurd (the number of possible, maybe useful, comparisons to include in the family may be infinite in cognitive science): clearly, how to choose a statistical threshold to decide whether an effect is “significant” or not is an ill-posed problem. I am not going to solve this problem here but I consider that “significant” results should be qualified given the *a priori* used to obtain them. Bayesian intuition (http://xkcd.com/1132/) interferes with NHST when not acknowledged (Dienes, 2011).

### An alternative to NHST

Like others (e.g., Cumming, 2013), I consider that publication should prefer confidence intervals to arbitrary significance threshold, in order to allow cumulative science (Yarkoni et al., 2010) rather than trying to reach conclusions after each study (CIs do not allow any probability statement on the population: a 95% CI means that, when repeating the experiment, 95% of the samples will include the true value within their 95% CI. This does not mean that the true effect lies between the bounds of a given 95% CI with 95% probability. Conclusions should therefore wait for meta-analyses: Cumming, 2012). In MRI, though, this may not always be easy or feasible given the thousands of comparisons made at each voxel. Improved solutions do exist to avoid the emphasis on dichotomous thinking based on an arbitrary threshold, which cannot be computed “correctly” anyway (Jernigan et al., 2003; Allen et al., 2012; ideally, results should be presented on the flat reconstructed cortical surface, or those in 3D should be available as an interactive, online, resource for every MRI study, like at http://neurovault.org/). Methods to plot the spatial distribution of confidence intervals over the brain have been proposed (Engel and Burton, 2013; Rosenblatt and Benjamini, 2014).

## Common mistakes with statistical inference

Neuroimaging a large cohort of subjects is difficult especially when having to recruit synesthetes, so the question of the minimum number of subjects required is crucial. Sinke et al. (2012) reminded us “that at least 12 subjects should participate in a fMRI group study (Desmond and Glover, 2002) but high reliability and sensitivity will only be achieved with more than 20 subjects” (Desmond and Glover, 2002; Thirion et al., 2007). These numbers may even be too low, especially for structural studies where the brain differences between two groups of healthy controls vs. synesthetes if any may be subtle. Most studies in neuroimaging (Yarkoni et al., 2010) and even neurosciences (Button et al., 2013) are underpowered. What “underpowered” means is not so clear, because this depends on the (unknown) size of the effects studied. Presenting data with confidence intervals rather than *p*-values (see previous paragraph) directly indicates to the reader the precision of the estimation, who can thus evaluate directly the power of the study (and therefore whether enough subjects were tested). However, since most MRI studies on synesthesia used small or even very small sample sizes, as well as the NHST logic, I first consider what should be the absolute minimal size for a “group” analysis, and then identify cases where the NHST logic, which may not be the optimal way to reach scientific conclusions (Meehl, 1967; Cohen, 1994; Kline, 2004; Cumming, 2012; Lambdin, 2012), is not even correctly applied. In particular, all computations depend on making the Null hypothesis, yet sometimes authors do not really make it or do not clearly define it.

### Sample size weakness

I relied on intuitive considerations in order to evaluate the results of studies based on very small numbers of subjects.

- If we consider two conditions (for example the intensity of the BOLD signal for two stimuli) and have too few measurements to quantitatively interpret the difference of signal^3^, we may still look at the sign of the difference. If the BOLD signal is *always* larger for a stimulus than the other, this is certainly meaningful. The most simple and robust way to evaluate “always” against chance is the sign test. This is equivalent to tossing a coin many times. The chance of always getting heads (or tails) is 7.3% when flipping a coin 5 times and 4.1% for 6 times (two-tailed test). Our usual, arbitrary, threshold being 5%, 6 should be the absolute minimum number of observations (or subjects) to be able to measure any “significant” effect; at least 7 subjects are required to verify that the result does not depend on any critical value (leave-one-out crossvalidation). Studies with less than 6 subjects should be treated as single-subject studies, and therefore the results of each subject should be shown (no group average).
- When comparing two groups of subjects on a given measure, what is the minimum number of subjects to be able to observe a “significant” difference? If the values in one group are all above the values in the other group, non-parametric Mann-Whitney gives a p-value below 0.05 only when groups include at least 4 subjects (*p* = 0.03). With 4 subjects no cross-validation at all is possible. The minimum size to reach half-split reliability is 8 subjects in each group. Group comparisons between 4 and 8 should, therefore, be treated with caution.

### Accepting the null hypothesis error

NHST only permit rejecting the Null hypothesis with some confidence, they do not provide any criterion for accepting the Null hypothesis. This is well known yet such error is often made when comparing the significance of tests performed independently in two groups (Nieuwenhuis et al., 2011), for example when comparing statistical maps in synesthetes vs. controls. Finding significant activations in synesthetes but not controls for a given contrast does not allow the conclusion that these activations are significant only in synesthetes. A direct comparison is required (typically testing the interaction between stimuli and group).

### Double dipping circular error

Computing the FWE over all brain voxels when one is only interested in a specific brain region increases the risk of Type II error. A common practice to increase power is to use *a priori* information, for example a region of interest (ROI), which allows reduction of the number of meaningful comparisons (reduced family or set size). But a circular error is made when using the same data to choose the “interesting” voxels and to test them (Kriegeskorte et al., 2009, 2010; Vul et al., 2009): the so-called *a priori* information is, in fact, defined *a posteriori*.

### Null hypothesis error (a hypothesis is not an a priori)

When wanting to use *a priori* information to decrease the set size and increase power, many studies mistook their *hypothesis* for an *a priori* (Hupé et al., 2012b). For example, assuming that “color area” V4 is activated by synesthetic colors is a reasonable hypothesis, leading authors to apply “small volume correction,” that is, only correcting their *p*-values by the number of voxels in the vicinity of V4. By doing so, however, they cannot suggest that V4 is activated by synesthetic colors, since the correct *description* of their *reasoning* is: “if it is hypothesized that voxels in V4 are activated by synesthetic colors (this is the hypothesis that led to restricting the Null hypothesis to V4), then voxels in V4 are observed with ‘significant’ activation.” This leads to some circularity: the activation is detected only if one supposed it exists, which means not making the whole brain Null hypothesis. The correct *interpretation* of their *analysis* is: “if we assume that synesthetic colors must activate V4, then we can identify which voxels if any within V4 are most likely to be activated by synesthetic colors.” This analysis can therefore be meaningful, especially if no “significant” voxel is found, but its description must include the conditional probability. *Showing* that some voxels in V4 are indeed activated by synesthetic colors requires one to make the Null hypothesis, that is, making the hypothesis that no voxels in V4 are activated by synesthetic colors. This Null hypothesis is not compatible with the restriction of the family of relevant inferences to the V4 region (unless stating that no activation by synesthetic colors is possible anywhere else in the brain^4^).

### Random vs. fixed effect

Ideally, we are interested in generalizing an effect observed in a sample of subjects to the population. To do that we consider that differences between subjects are random variations. Other subjects could have been tested (the choice of subjects is supposed to be random). When computing only one summary measure by subject the only measured variance to compute NHST is across-subject variability, and “subject” is, therefore, a random variable. But often, several measures (repetitions) are computed by subject, leading to within-subject variability in addition to across-subject variability. One may be interested in the differences between the chosen subjects. In that case, one can contrast across-subject variability against the pooled within-subjects variability (noise term). This is called a fixed-effect analysis (if you want to replicate the analysis you should test the same subjects^5^). If two conditions were tested (fixed effect), results across subjects apply only to the tested sample. In order to be able to generalize to the population, across-subject variability needs to be included in the noise term by specifying in the statistical design that “subject” is a random variable (“mixed model,” which includes both fixed and random effects). In complex data analyses, in particular, whether subject variability is taken as a random factor is not always clear, for example when a network analysis requires computing one single statistics for a group of subjects.

### Selective reporting

In several studies interesting comparisons were planned, as can be deduced from the Methods section. Unfortunately in some studies only selective results are then reported. Non-reported results, maybe not consistent with the main message in the paper, could, however, be informative to the community. Selective reporting practices can sometimes be detected when too many “just significant” results are published (e.g., Francis, 2012).

## Pitfalls of MRI statistics

The analysis of MRI data requires specific models that go beyond the simple principles of statistics described above, in order to address two major problems.

1. MRI measures information locally (within each voxel) over the whole brain. A voxel is not a functional unit. On one hand, each voxel contains thousands of neurons; on the other hand, functional or structural information may be distributed over several voxels: measures across voxels are not independent, but to an unknown, experiment dependent, degree. This makes difficult the proper control of the inflated risk of false positives across many voxels.
2. Brains are different so the measure in corresponding voxels across subjects may not sample comparable information. A fundamental problem is what information is being matched between brains; the thorough discussion and possible resolutions of this problem is beyond the scope of the present paper.

Here I describe the logic of the statistical models used in the reviewed papers (Hupé and Dojat, 2015), again the way I understand it as a “statistics-aware” MRI user, not a statistician.

### Regions of interest

When possible, a powerful method to match information between brains is to identify functional units that are similar in each brain (e.g., Poldrack, 2007). For example, retinotopic mapping allows identification of (at least) visual areas V1 to V4 in each subject with some confidence. Signals can then be measured in each of these regions of interest (ROI) and compared across subjects. A related approach is the use of functional localizers to identify brain regions that respond more to motion or color (for example). A problem arises when there is a lack of strict correspondence between structure and function (for example, there is no single “color” region, and this is certainly not strictly retinotopic V4: Brewer et al., 2005; Hupé et al., 2012c), or when the protocol may not unambiguously identify a functional area (for example, the classical Mondrian localizer for color areas lacks specificity related to color processes; moreover, the definition of the ROI requires an arbitrary threshold, which leads to make an inference error of the type “Accepting the Null hypothesis error”; see Jernigan et al., 2003). The ROI approach is, therefore, interesting and powerful but the results depend on the hypotheses made to define and identify the ROIs, which may involve questionable choices when done beyond retinotopic areas.

### Random field theory: peak statistics

*Voxelwise comparisons* across subjects do not rely on such hypotheses and choices, but directly face the two major problems of brain differences and performing thousands of comparisons. The solution to structural differences, spatial smoothing and transforming each brain to a common space, would be correct only if we had exactly the same brain except for some linear (or even non-linear) scaling factors. Inferences based on across-subjects statistics depend on how wrong this approximation is. To address the problem of multiple comparisons, the random field theory (RFT) takes into account correlations over neighboring voxels to control the risk of false positives over the whole brain (Worsley et al., 1992). RFT is applied to statistical maps, for example the difference of BOLD signal in each voxel measured for two different stimuli, typically expressed as a *t*-value or z-score (Friston et al., 1995). RFT estimates the smoothness (spatial correlations) and variance of the statistical map in order to approximate the upper tail of the maximal distribution of the statistics: it computes the t or z threshold above which there is less than say 5% chance of observing one cluster of voxels with values above that threshold, under the Null hypothesis^6^. These “peak statistics” require several assumptions to be exact (Petersson et al., 1999; Nichols and Hayasaka, 2003), in particular the “reasonable lattice approximation,” which is obtained when data have been sufficiently spatially smoothed and the distribution of errors (across trials or subjects) is Gaussian. A major issue for this kind of multivariate analysis is the spatial heterogeneity of variance across the brain (“non-stationarity”), especially for structural data (Ashburner and Friston, 2000).

The measure of peak statistics using the RFT, to be optimal (powerful), requires that the spatial filter used to smooth the data be about the same size as the spatial extent of the effect to be measured. This extent is typically unknown and can be very different depending on what is measured and where in the brain. Even if a functional activation is very specific and is localized at the exact same anatomical region in each brain (for example the depth of a given sulcus), if much anatomical variability of this sulcus exists across subjects (even after normalization to a common standard space) this activation could reveal as significant across subjects only when applying a very large spatial filter (note that the localization of the effect would be less precise). This consideration led Poline and Mazoyer (1994) to propose a multifiltering approach. This method has not been pursued because it required large computer resources, a problem now obsolete, even though it was efficient and robust (Poline et al., 1997) and relied on the theoretically strong RFT. It also introduced a new problem of multiple comparisons (the number of “independent” filter sizes) as well as overfitting (such an approach may fit the spatial filter to random noise in the data).

### Random field theory: cluster extent statistics

*Cluster extent statistics* is an alternative strategy to voxelwise statistics, now widely used, which somehow addresses the same issue as the multifiltering approach. Using RFT, it is possible to compute *the number k of voxels in a cluster*, all with values above a given t or z threshold, beyond which there is less than say 5% chance of observing a cluster, under the Null hypothesis (Poline and Mazoyer, 1993). Such computation requires deciding on an arbitrary threshold, but then controls the risk of false positives over the whole brain. Most MRI studies now report this cluster extent statistics, which is typically more sensitive than voxelwise statistics to revealing significant effects. The interpretation of the effects, however, is not as straightforward as for voxelwise statistics, because the inference concerns “having k contiguous voxels above a given threshold” (in other terms, nothing can be said about specific subregions of the cluster; yet most reviewed papers, including ours, only reported one voxel coordinate). Such an effect could be obtained, for example, if all subjects have a weak but similar activation all over the visual cortex (a weak but widespread effect in each subject). But significant clusters can also emerge for highly focal activations but differently localized in each subject, like obtained when contrasting colored against greyscale Mondrian stimuli (peak activations are observed in each subject within the same region, but with much variability in the precise anatomical location and number of peaks: Brewer et al., 2005; Hupé et al., 2012b,c). Whatever its interpretation, the validity of cluster extent statistics depends crucially on spatial smoothing and the chosen threshold, so these values should be systematically reported (in our review we reported them in the summary of each study).

Contrary to parametric tests of the central tendency (like the ANOVA), statistics of maximal values (peak or cluster extent) are very sensitive to deviations from their conditions of validity, like unequal variance and extreme values (outliers). Empirical distributions (obtained with data permutations) are indeed highly skewed, especially for cluster extent (see Figure 1 by Hayasaka and Nichols, 2004), making the estimation of the upper tail very sensitive to such deviations. Much effort has been devoted, therefore, to produce a Gaussian distribution of the statistics at each voxel and minimize the impact of non-stationarity. When the roughness/smoothness of images was poorly estimated, *p*-values were shown to be up to ±20% inaccurate (Poline et al., 1995). Improved methods include, for example, smoothness estimation from standardized residual images (Kiebel et al., 1999), or weighting by the variance in each group, even under deviation from normality (Behrens Fisher problem), using Brunner Munzel statistics (Brunner and Munzel, 2000; Neubert and Brunner, 2007; Rorden et al., 2007). In classical ANOVA the conditions of validity are easily checked by examination of residuals, which is more difficult with multivariate analysis. A method is implemented in SPM^7^ “Distance” toolbox (Kherif et al., 2003) to visualize the multivariate distribution of residuals and identify possible outliers. Note however that the identification of “true” outliers can be obtained only with large data sets, like *N* > 30. Rejecting outliers based on small populations like used in MRI studies may lead to the rejection of valid observations and therefore fitting the data to the model instead of fitting the model to the data (data identified as outliers could in any case be rejected only for an independent, valid, reason that can apply to the whole sample). Inspection of residuals is rarely reported in MRI studies and never in the papers that we reviewed.

Non-stationarity also causes the reasonable lattice approximation to break down at low thresholds of statistical values (high *p*-values). For cluster-extent statistics, t or z-statistics thresholds should therefore be at least above 3 (Poline et al., 1997) or 4 (Smith and Nichols, 2009; see also Woo et al., 2014). Non-stationarity had also led Ashburner and Friston (2000) to discourage cluster-size statistics for VBM data. However, simulations based on empirical data show that a large degree of spatial smoothing is indeed necessary but also usually sufficient to obtain reliable results (this procedure also weakens the weight of large or extreme values measured locally in only a few subjects), at the cost of the precision of localization of effects. Thus, Silver et al. (2011) recommended that “cluster size inference should only be used with high cluster-forming thresholds and smoothness”, such as *p* = 0.001 for voxel threshold and a 12 mm Gaussian kernel (Full Width at Half Maximum, FWHM). They observed in simulations that “false positive rates ranged from 9.8 to 67.6%” when using a 6 mm Gaussian kernel and thresholds such as *p* = 0.05 or *p* = 0.01. The required spatial smoothing may be different for VBM (including diffusion anisotropy, DTI) and FMRI, and must also depend on each study. I don't know of any study reporting a measure of stationarity in their data, and whether any tool is available.

Even when applied under optimal conditions, cluster extent statistics pose several problems. Nichols acknowledged knowing “of no formal proof that cluster inference has such strong control of Familywise error” (Nichols, 2012), while Smith and Nichols (2009) suggested that it may be “hard to persuade the experimenter to honestly correct for “multiple comparisons” across different thresholdings.” I consider indeed that this statistics faces the same problems as the multifiltering approach: multiple testing and possible overfitting^8^. It also poses the problem of selective reporting and comparison between studies. Usage wants authors reporting only the value of the cluster-forming threshold. But did they try other thresholds? Are results different at other thresholds, for example could other “significant” clusters be discovered that may be more difficult to explain? When authors report only voxelwise statistics (for example no significant difference between two conditions or two groups) did they also compute cluster extent statistics and observed no significant cluster? While visualization of effects rather than significance maps may solve this problem in the future (Allen et al., 2012), for the present studies, meta-analysis faces strong limitations.

### Permutation tests

While SPM software is relying mostly on RFT and parametric computations, FSL^9^ software favors permutation tests, which can be applied to voxel maximum *t*- or *z*-value and cluster extent statistics. Permutation tests are elegant because they only require the assumption of exchangeability. However, the nature of the inference also depends on this assumption. If nothing else is known, the only conclusion based on a “significant” permutation test is that exchangeability is violated—that is, two groups are different. However, one cannot infer what the nature of the difference is. This is the case, for example, for the classic Wilcoxon test, a permutation test based on ranks (Manly, 1997). This test is typically used as an alternative to the parametric *t*-test when normality is violated. However, like the *t*-test, valid inference on the central tendency (mean or median) is only guarantied when the variances are similar (homoscedasticity hypothesis). Permutation tests are also sensitive to outliers. Imagine that when comparing a group of 20 synesthetes to a group of 20 controls using a given statistic, 5 subjects are clearly outliers (larger values) to a normal distribution of this statistic, all of them synesthetes. These values drive a larger summary value across synesthetes than controls. By using a permutation test you do not assume normality so you would not identify (and possibly exclude) these subjects. You may well observe that the larger value across synesthetes can hardly be due to chance—the permutation test would mostly compute the chance of having the 5 extreme values all within the same group, which is 0.5^∧ 5^ = 0.03. You would, therefore, conclude that the two groups are different. Such a conclusion is correct but the interpretation would be wrong if you conclude that a correlate of synesthesia is a larger value for your measure. Such an interpretation would be based on a model of the type: empirical observation = true value + error. This model is clearly wrong here. The correct interpretation is that the group of synesthetes is more likely to include outliers for this measure. In fact, this fictitious example could happen quite easily in case of comorbidity, as suggested for the higher rate of radiologically determined white matter hyperintensities (one of the imaging criteria for the diagnosis of multiple sclerosis) in self-referred synesthetes who had participated in neuroimaging research (Simner et al., 2014). The correct interpretation should be the presence of comorbidity in the tested sample, but this would tell nothing about the correlates of synesthesia. In most cases, of course, the results of permutation tests do not depend so strongly on outliers (and in the example above, at most one such case was observed in any single study, and when identified, could be excluded from the analysis). But each time one wants to infer about the central tendency of an effect, permutation tests provide statistical measures that are inexact to an unknown degree. Only with “everything else being equal” can we make strong inferences on the central tendency. In that case, parametric statistics (when possible) should provide the exact same results. Parametric models are often more powerful when they include covariates (like blinks in fMRI studies or brain size in VBM studies)^10^.

### False discovery rate

All the statistical measures described above were developed to control the risk of being wrong when rejecting the Null hypothesis. An alternative is the computation of the false discovery rate (FDR), the expected proportion of false positives among detections (Benjamini and Hochberg, 1995). This test has the great advantage of providing meaningful results even when multiple tests are not independent, like across voxels. This also provides two-tailed statistics on the central tendency and does not rely on the unstable estimation of the upper tail of the statistics (when applied voxelwise). However, similarly to Bayesian statistics, FDR results depend on the probability of non-Null effects: measured FDR *p*-values for an effect of interest depend on how often the Null hypothesis is non-true. This becomes a problem when many Null hypotheses are non-true *for the wrong reasons* or because the family of tests is too large (it includes tests that have little reason to be included in the “family”). Let's take a simple example: synesthetes may blink more often after synesthetic stimuli because they start thinking about the synesthetic color and they need to refocus on the task. Blinks activate a large portion of the visual cortex, mostly along the parieto-occipital cortex and the anterior calcarine, with only minimum influence on responses beyond V4 or in central V4 (Hupé et al., 2012a). Such a behavior may therefore not affect the central V4 responses to colored stimuli. However, this will affect the computation of the FDR value. This makes therefore the interpretation of the FDR value more problematic than FWE-corrected *p*-values, obtained under the Null hypothesis. As a consequence, sloppy designs may generate more easily “significant values”: FDR may “detect” the expected (“desired”) effect more easily when many differences unrelated to the question asked are present. This non-desirable behavior is counter-intuitive, since “sloppy” designs would be rather expected to increase variance (which they may also do, fortunately) and therefore decrease significance.

## Conclusion

I would like to be able to provide some recommendations on the best (or at least the less bad) way to analyze MRI data, but I am not qualified to do so. I should remind the reader that this text was not written by a statistician or a developer of MRI analysis. If recommendations should yet be done to users, the first one should be to better detail the analysis pipeline (Poldrack et al., 2008). The second one would be to try understanding better the tools used (like I strove here), to be aware of their strong limitations, and be suspicious of “hypothesis-free” solutions (like FDR or permutation tests). The third recommendation would be data sharing (Poline et al., 2012; Poldrack et al., 2013). But recommendations should also be directed to developers of MRI tools. After decades of *p*-value diktat (Meehl, 1967; Cohen, 1994; Kline, 2004; Lambdin, 2012), psychologists may be at last ready to switch to Confidence Intervals, thanks in particular to the success of the pedagogical effort by Cumming (2012, 2013). The challenge will be to apply the Confidence Interval logic to the huge and complex data sets of brain imaging studies.

### Conflict of interest statement

The author declares that the research was conducted in the absence of any commercial or financial relationships that could be construed as a potential conflict of interest.

## Footnotes

^1^The mean of the log data is therefore also the median and the mode of the distribution of the transformed data; it is called the “location” parameter of the lognormal distribution. Most synesthesia studies of response times, RT, (e.g., synesthetic Stroop tests) computed the mean of non-transformed RT as summary measures; moreover, they often used invalid criteria to exclude so-called “outliers,” values larger than 2 or 3 standard deviations; such definition of an outlier supposed the RT distribution to be Gaussian (and even if it was the case this would not be a sufficient reason to exclude these values). Conclusions based on NHST and close to the decided significance threshold may well be sensitive to such incorrect procedures. Classical transformations for RTs are lognormal and inverse. “Transformation” of the data should be understood as the way to apply a lognormal or inverse model.

^2^Note that the better model could be as simple as deciding of cutoff criteria, for example the exclusion of very short and very long response times; but this would not be a statistical criterion; the author would have to justify that too short or too long RTs are not possible values reflecting the task; and such exclusion criterion may well exclude values that were not detected as outliers. Here, we are not dealing with the cases when the “true” distribution is known and yet estimators, like central tendency measures, may be biased because of a few extreme values; these cases may be dealt with robust statistics (e.g., Pernet et al., 2012).

^3^It would be convenient to be able to propose a value for ≪ too few ≫. Simmons et al. (2012) did request that “authors must collect at least 20 observations per cell.” This value was based on simulation, not on theoretical grounds. It is also related to power and decision criteria based on NHST. Here, the question concerns the meaning of quantitative values. At the very beginning of this paper, I reminded that “quantitative measures assume a model of the form: empirical observation = true value + error.” We learn in mathematics class that the Central Limit Theorem states that the sample mean follows a normal distribution when the sample is drawn randomly and the sample size is large enough, 30 being considered as large enough. However, I am not aware of any theoretical justification for this number. This may yet be a reasonable rule of thumb; based on my own experience, it is very difficult to verify whether a distribution may be close enough to the normal distribution with fewer than 30 values.

^4^Let's be very clear about that: I am not arguing against the use of small volume correction (SVC) or region of interest (ROI) analysis (ROI analyses do not need to use NHST: they may simply show Confidence Intervals). I am only pleading for awareness of the Null hypothesis as well as consistency. Small volume correction determines a *t*- or *z*-score above which voxels are considered as significant. When data is available this threshold should be applied to the whole brain. If voxels outside the small volume are above this threshold this means that the restriction of the family of tests was not justified (often studies reported such unexpected activation while maintaining the conclusion based on SVC). Likewise, ROI analyses should be completed by whole brain analyses to avoid pinhole conclusions.

^5^A significant main effect of the variable “subject” means that across-subjects differences are not only due to sampling error. Possible interactions between the tested effect and “subject” should then be examined. I do not know of any MRI study that considered subject variability that way. In the rare cases of fixed-effect analyses across-subject variability was simply factored out, implicitly making the very strong assumption that the measured variable could not be influenced by subject variability (as if a single “supersubject” had run the whole experiment).

^6^Estimation of the upper-tail distribution is by definition a one-tailed test. This is correct for the statistics of interactions (*F*-test), but testing differences between two populations requires performing two tests. Under the Null hypothesis, and, therefore, not assuming the direction of the effect, all reported effects obtained at “p = 0.05” should, therefore, be reconsidered as “p = 0.10” for a two-tailed test.

^7^http://www.fil.ion.ucl.ac.uk/spm/

^8^The “threshold-free cluster enhancement” statistics (TFCE, Smith and Nichols, 2009), implemented in FSL, somehow overcomes this problem, by proposing a single statistic. It however requires deciding on two parameters, instead on only one (cluster defining threshold). Values for these parameters are proposed by default in FSL that are supposed to be appropriate for most studies (Smith and Nichols, 2009).

^9^http://fsl.fmrib.ox.ac.uk/fsl/fslwiki/.

^10^Usage of covariates in permutation tests for MRI seems rare. In addition, the correct estimation of the upper-tail distribution (for peak or cluster extent statistics) with Monte-Carlo stimulations or permutations requires a very large number of samples or permutations, especially for highly skewed statistics on cluster extent.
